# Serum Vitamin E Levels in Multiple Sclerosis: Association with Diagnosis, Cognitive Function, Disability, and Depressive Symptoms

**DOI:** 10.1007/s12035-026-05878-w

**Published:** 2026-04-30

**Authors:** Francesco Bruno, Patrizia Spadafora, Mario Luca Cuconati, Antonio Qualtieri, Ida Veltri, Selene De Benedittis, Beatrice Maria Greco, Annamaria Cerantonio, Luigi Citrigno, Gemma Di Palma, Olivier Gallo, Alberto Montesanto, Francesca Cavalcanti

**Affiliations:** 1 Department of Human and Social Sciences, Faculty of Social and Communication Sciences, Universitas Mercatorum, Piazza Mattei 10, 00186 Rome, Italy; 2 Institute for Biomedical Research and Innovation (IRIB), Italian National Research Council (CNR), Loc. Burga 44, 87050 Mangone, CS Italy; 3 Student at the Department of Medical and Surgical Sciences, Science and Techniques of Cognitive Psychology Degree Course, Magna Graecia University of Catanzaro, Viale Europa Snc, 88100 Catanzaro, Italy; 4 Territorial Social-Health Company of Lodi, Piazza Ospitale 10, 26900 Lodi, Italy; 5 Department of Biology, Ecology and Earth Sciences, University of Calabria, Via Pietro Bucci Snc, 87036 Rende, CS Italy

**Keywords:** Multiple sclerosis, Vitamin E, Cognitive function, Disability, Depressive symptoms, Oxidative stress

## Abstract

Multiple sclerosis (MS) is a chronic inflammatory and neurodegenerative disease of the central nervous system and the most common neurodegenerative disease in young adults, characterized by motor, sensory, visual, and cognitive impairments. Vitamin E (α-Tocopherol), a fat-soluble antioxidant, may influence cognitive function, disability, and mood in MS. One hundred eighty-four Italian Caucasian MS patients and ninety-four healthy controls were included. Clinical and demographic variables were recorded, and serum vitamin E levels were measured by HPLC. Cognitive function was assessed using the Mini-Mental State Examination (MMSE), disability with the Expanded Disability Status Scale (EDSS), and depressive symptoms with the Hamilton Depression Rating Scale (HDRS). Logistic and linear regression analyses were performed, adjusting for demographic, MS-related, and metabolic confounders. Bonferroni correction was applied. Discriminative ability for clinical cut-offs (MMSE ≤ 24, EDSS ≥ 6, HDRS ≥ 8) was assessed using receiver operating characteristic (ROC) curves. MS patients had significantly lower serum vitamin E levels compared to controls. Lower vitamin E levels were associated with MS diagnosis. In MS patients, higher vitamin E levels were associated with better cognitive performance and lower disability. All associations remained significant after Bonferroni correction in fully adjusted models. No significant relationship was found between vitamin E levels and depressive symptoms. Vitamin E showed good discriminative ability. Serum vitamin E levels showed significant associations with MS diagnosis, cognitive function, and disability, with good discriminative ability. Longitudinal studies are needed to clarify whether low vitamin E levels influence the risk of developing MS, while randomized controlled trials are warranted to determine whether vitamin E supplementation may provide clinical benefits.

**Supplementary Information:**

The online version contains supplementary material available at 10.1007/s12035-026-05878-w.

## Introduction

Multiple sclerosis (MS) is a chronic inflammatory, autoimmune, and neurodegenerative disease of the central nervous system (CNS), with a complex and variable clinical presentation. It primarily affects young adults, with a peak onset between the ages of 20 and 40, and is more common in females than males, with a female-to-male ratio of approximately 3:1 [1, 2]. MS is characterized by demyelination, axonal damage, and subsequent neurodegeneration, leading to a range of neurological symptoms [3]. These symptoms are heterogeneous, with patients presenting with a variety of clinical manifestations, including motor, sensory, visual, and cognitive impairments as well as mood disorders [4–6]. MS is classified into several subtypes, including relapsing–remitting (RR) MS, primary progressive MS (PPMS), secondary progressive MS (SPMS), and clinically isolated syndrome (CIS), each associated with different patterns of disease progression and clinical outcomes [7].

One of the most debilitating aspects of MS is its impact on cognitive function, with prevalence rates ranging from 43 to 70% [8]. Cognitive dysfunction in MS typically involves deficits in attention, memory, information processing speed, and executive function. These cognitive impairments can significantly reduce the quality of life, impair work functioning, and affect social interactions, often leading to increased disability and a greater burden on patients and caregivers. In addition to cognitive dysfunction, depression is a common comorbidity in MS, affecting approximately 60% of patients [8]. Depression in MS is multifactorial, influenced by both biological factors, such as the neuroinflammatory processes and neurodegeneration associated with MS, as well as psychosocial factors like the chronicity of the disease and the associated disability [9]. Depression in MS can exacerbate disability and cognitive decline, creating a vicious cycle that further worsens the patient's clinical outcomes [10]. Disability in MS is typically measured using the Expanded Disability Status Scale (EDSS), which quantifies the degree of impairment across different functional domains, such as mobility, visual function, and cognitive status [11]. Disability progression in MS is variable, with some individuals remaining relatively stable for many years, while others experience rapid deterioration. The factors influencing disease progression and disability are not yet fully understood, but they likely involve a combination of genetic, immune-mediated damage, neurodegeneration, and oxidative stress [12].

One potential modifiable factor that may influence cognitive function, disability, and mood in MS is vitamin E (α-Tocopherol). Vitamin E is a fat-soluble antioxidant with well-documented properties in protecting cells from oxidative stress, which is an imbalance between reactive oxygen species (ROS) and the body’s antioxidant defenses [13]. Oxidative stress is thought to play a central role in the pathophysiology of MS, contributing to the demyelination of axons, neuronal damage, and disease progression [14, 15]. The neuroprotective effects of vitamin E are attributed to its ability to scavenge free radicals and reduce lipid peroxidation, thereby protecting cellular membranes, including those in the brain and spinal cord, from oxidative damage [16]. In addition to its antioxidant properties, vitamin E is involved in immune modulation and may play a role in reducing neuroinflammation, a hallmark of MS pathology [17, 18]. Several studies have suggested that oxidative stress contributes not only to the physical symptoms of MS but also to cognitive decline and the development of depression [19, 20]. Increased oxidative damage can impair neuronal function, leading to cognitive deficits and exacerbating disability [21]. Furthermore, oxidative stress has been implicated in the pathophysiology of mood disorders, including depression, by affecting serotonin and other neurotransmitter systems involved in mood regulation [22]. Research has shown that serum vitamin E levels and the vitamin E/cholesterol ratio are significantly lower in MS patients compared to controls, while cerebrospinal fluid (CSF) vitamin E levels remain unchanged [23–25]. Plasma vitamin E levels decrease during MS exacerbations and increase with IFN-beta treatment, likely reflecting disease suppression and oxidative stress. Animal studies suggest vitamin E can protect against demyelination and enhance remyelination, and may have therapeutic potential by inhibiting the NF-kB pathway which is involved in MS pathology [26–29]. Considering the role of vitamin E in cognitive function, disability, and depression, it does not appear that such associations have been thoroughly explored in previous studies. Therefore, the purpose of this study is to investigate the potential links between serum vitamin E levels and these key aspects of MS. In particular, we seek to determine whether vitamin E levels are associated with the MS diagnosis, cognitive function, disability, and depressive symptoms, while controlling for confounding factors such as demographic variables (i.e., age, sex, education), MS-related factors (e.g., disease duration, MS subtype, relapse history, and use of disease-modifying therapies), and conditions that could influence serum vitamin E levels (e.g., thyroid disorders, hypertension, smoking status, alcohol intake, and Body Mass Index; BMI). Additionally, we investigate whether vitamin E levels discriminate between patients with cognitive impairment, depressive symptoms and higher disability, using well-established clinical cut-offs.

## Materials and Methods

### Patients

The study included Italian patients with MS, who were followed at the National Research Council (CNR)—Institute for Biomedical Research and Innovation (IRIB) in Mangone, Cosenza, Italy. All participants underwent a comprehensive clinical evaluation to confirm the diagnosis of MS. Patients had been originally diagnosed according to Poser's criteria [30]; however, clinical and neuroradiological review confirmed that all cases fulfilled the 2017 McDonald criteria [31]. Data were retrospectively retrieved from their medical records, provided the clinical information was complete. The inclusion criteria were as follows: (i) age over 18 years; (ii) diagnosis of MS according to the 2017 revised McDonald criteria [31]; (iii) availability of the Mini-Mental State Examination (MMSE) [32], Expanded Disability Status Scale (EDSS), and Hamilton Depression Rating Scale (HDRS) scores [11, 33, 34]; (iv) no history of head trauma, neurological conditions other than MS, intellectual disability, or serious medical conditions; and (v) availability of a blood sample. The following conditions were exclusion criteria: i) presence of other neurological disorders; ii) a history of head trauma; iii) major psychiatric illnesses; iv) visual deficits or upper limb motor impairment that could interfere with the cognitive and mood assessments and; v) use of medications that could affect cognitive performance. The following clinical and demographic variables were recorded for each patient: sex, age, years of education, MS subtype, age at disease onset, disease duration, relapse in the last year (yes/no), use of disease-modifying therapies (yes/no), general cognitive function according to the MMSE score, level of disability according to the EDSS score, severity of depressive symptoms according to the HDRS score. Additionally, factors that could potentially influence serum vitamin E levels were recorded for all patients: presence of thyroid disorders, hypertension, hypercholesterolemia, type II diabetes mellitus, smoking status, alcohol intake, and BMI. The research was conducted in accordance with the Helsinki Declaration of 1975 and its subsequent amendments. Ethical approval was not required, as the study utilized non-identifiable data and anonymized biological materials, in line with local regulations. Written informed consent was obtained from all participants for the blood sample collection and clinical data.

### Sample Preparation

The classical procedure for the extraction of lipophilic vitamins from serum samples was used with a few modifications [35]. Briefly, 100 l of serum was mixed with an equal volume of ethanol and vortex vigorously for 10 s. Subsequently, 100 l of n-hexane was added, and the mixture was shaken for 45 s and centrifuged at 800 g for 5 min. The upper organic layer, approximately 75 l, was transferred to a new tube and dried under low pressure with a SpeedVac. The residue was then redissolved with 100 l of a 75:25 methanol-ethyl acetate mixture and 50 l was injected into the chromatographic system.

### HPLC Separation

**Fig. 1:**
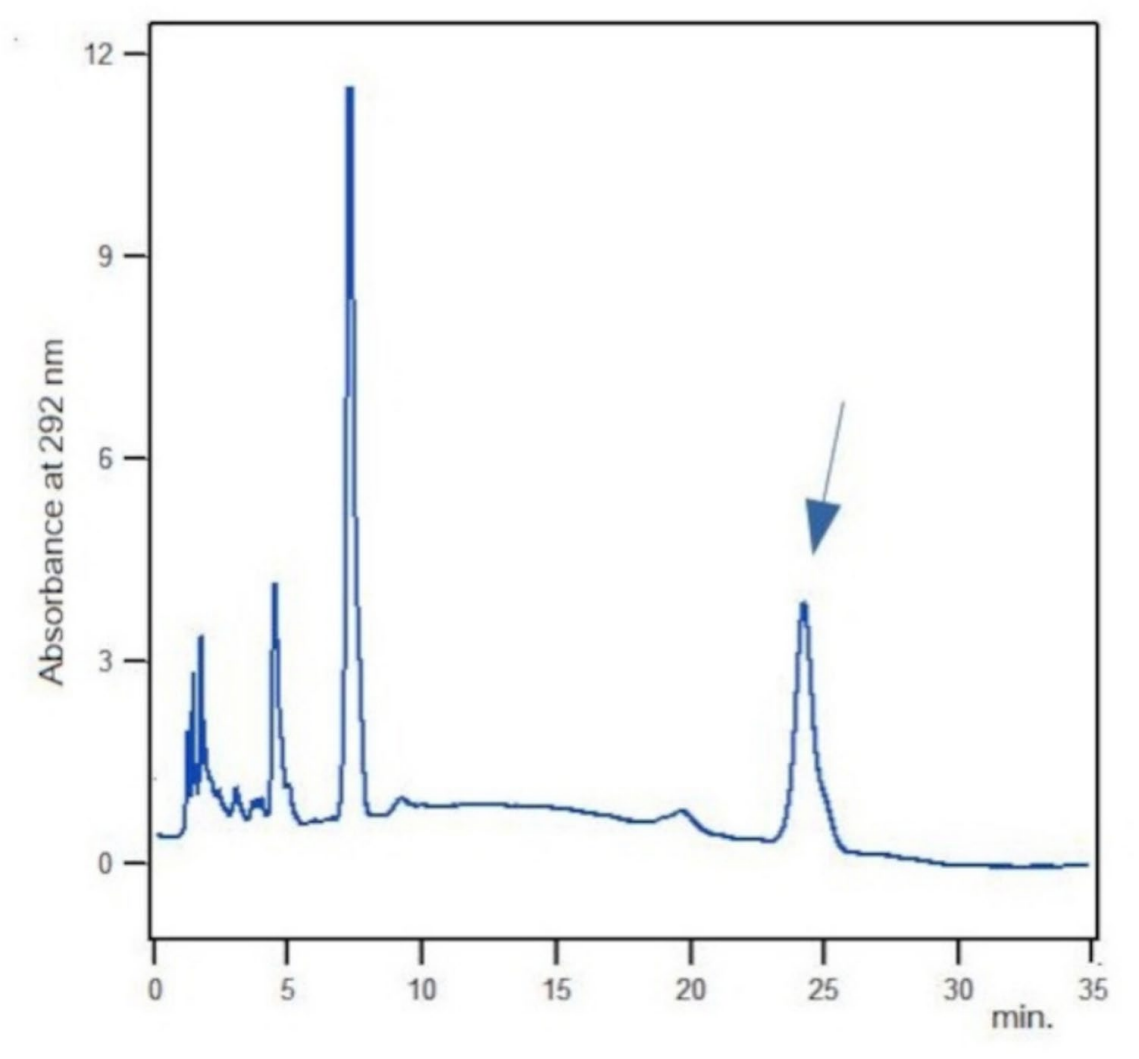
A representative HPLC chromatogram of a control sample. Arrow indicates the a-tocopherol peak

An Agilent 1100 series HPLC system (Agilent, Santa Clara CA, USA) equipped with a G1314A VWD UV detector, a G1321A FLD detector and a G1312A binary pump was used. Separation was performed on C18 Vydac Genesis 150 × 4.6 mm, 5 columns with 95% methanol buffer eluted isocratically at 1 ml/min for 35 min and acquiring data at 292 nm. The peak area value of vitamin E from each run was recorded for quantitative analysis (Fig. 1).

### Standard and Calibration Curve

Standard solutions were prepared from a 50 mg/l stock solution of alpha-tocopherol in ethanol. Five different concentrations at 3.62, 7.25, 14.5, 29, 58 mol/l were prepared and used to construct a calibration curve. The average vit E value of two HPLC runs was calculated for each assay and used to determine the serum concentration using the five-point calibration curve.

## Statistical Analyses

Data were analyzed using Jamovi software (version 2.3.18). Descriptive statistics were performed on demographic and clinical characteristics. Means and standard deviations (M ± SD) were calculated for continuous variables, and frequencies and percentages (n/%) for categorical variables. Logistic regression analyses were used to estimate how the serum vitamin E levels were associated with MS diagnosis. Analyses were unadjusted (Model 1) and corrected for sex and age (Model 2). Associations were presented as odds ratios (OR) and their corresponding Confidence Intervals (CI). Linear regression analyses were conducted to examine the association between MMSE, EDSS, and HDRS scores and serum levels of vitamin E adjusting for potential confounders. The first model did not include any covariates; the second model included age, sex, and education as covariates for MMSE and HDRS and age and sex as covariates for EDSS; the third model included additional MS-related covariates that could influence MMSE, EDSS, or HDRS scores (i.e., disease duration, MS subtype, relapse in the last year, use of disease-modifying drugs); the fourth model included all potential covariates that could influence serum vitamin E levels (i.e., hyperthyroidism, hypertension, smoking status, alcohol consumption, and BMI). The same analyses were replicated by fitting logistic regression models categorizing the dependent variables using the recognized clinical cut-off values of ≤ 24 for MMSE, ≥ 6 for EDSS, and ≥ 8 for HDRS [36–38]. Model performance was evaluated in terms of Area Under the Receiver-operating characteristic curve (AUC). Independent samples t-test was used to compare the serum vitamin E levels between MS patients and the control group, as well as between MS patients classified according to the clinical cut-offs. Normality of continuous variables and regression residuals was assessed using the Shapiro–Wilk test and visual inspection of histograms and Q–Q plots. Although the Shapiro–Wilk test was statistically significant in some instances, visual inspection indicated only minor deviations from normality, primarily in the distribution tails. Given the sample size, parametric tests and linear regression models were considered robust to moderate deviations from normality. A p-value < 0.05 was considered statistically significant; for the four primary outcomes (MS diagnosis, MMSE, EDSS, HDRS), Bonferroni correction was applied, setting the adjusted significance threshold at p < 0.0125 (0.05/4).

## Results

### Participants Characteristics

Descriptive statistics for demographic and clinical characteristics are shown in Table 1. The final sample consisted of 184 MS patients with a mean age of 36.71 ± 10.58 years. The majority of the patients was female (63.04%) and had RR-MS (70.66%), followed by PP-MS (13.04%), CIS (10.87%), and SP-MS (5.43%). The mean years of education were 11.03 ± 4.03, the mean age at MS onset was 29.10 ± 10.70 years, and the mean disease duration was 8.70 ± 7.84 years. The mean MMSE, EDSS, and HDRS scores were 26.90 ± 2.34, 1.80 ± 1.95, and 6.19 ± 4.50, respectively. Thirty-one subjects (16.8%) were smokers, and 8 (4.3%) consumed alcohol daily with meals. Regarding comorbidities, 3 patients (1.6%) had hypertension and 1 (0.54%) had hyperthyroidism. None of the patients included in the study had type II diabetes mellitus or hypercholesterolemia. Finally, the mean BMI was 25.06 ± 4.76.

**Table 1:** Demographic and clinical characteristics of patients (n = 184)

Age (years)	36.71 ± 10.58
Sex	
* Female*	116 (63.04)
* Male*	68 (36.96)
Education (years)	11.03 ± 4.03
Age of MS onset (years)	29.10 ± 10.70
Duration of MS (years)	8.70 ± 7.84
MS subtype	
CIS	20 (10.87)
RR	130 (70.66)
PP	24 (13.04)
SP	10 (5.43)
MMSE	26.90 ± 2.34
EDSS	1.80 ± 1.95
HDRS	6.19 ± 4.50
BMI	25.06 ± 4.76
Smoking status	31(16.8)
Alcohol intake	8 (4.35)
Type II Diabetes Mellitus	0 (0)
Hyperthyroidism	1(0.54)
Hypertension	3(1.63)
Hypercholesterolemia	0 (0)

Data are presented as mean ± Standard Deviation (m ± SD) or n (%). *CIS* Clinically Isolated Syndrome, *RR* Relapsing–Remitting, *PP* Primary Progressive, *SP* Secondary Progressive. *MMSE* Mini-Mental State Examination, *EDSS* Expanded Disability Status Scale, *HDRS* Hamilton Depression Rating Scale, *BMI* Body Mass Index

### MS Diagnosis

**Fig. 2:**
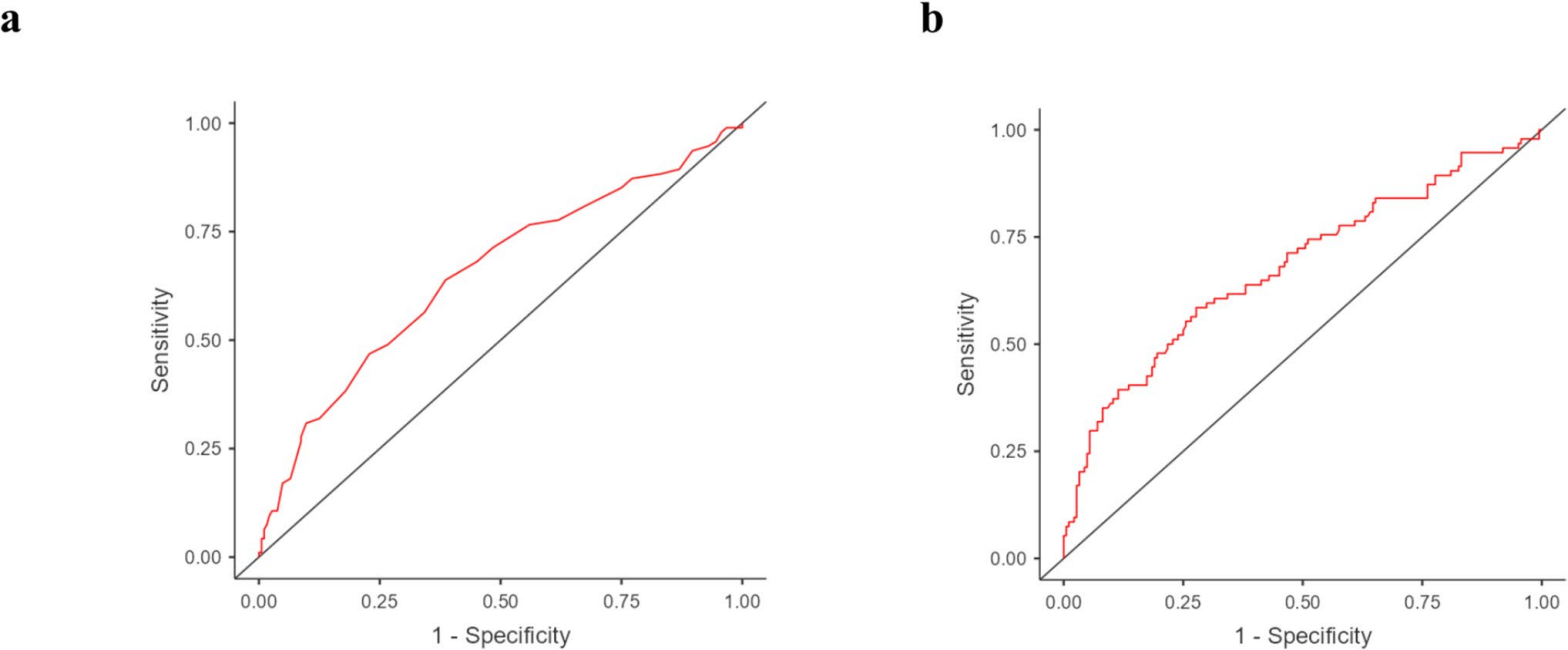
ROC curves for the two logistic regression models assessing the association between serum vitamin E levels and MS diagnosis. Panel A shows *Model 1* (unadjusted), while Panel B shows *Model 2* (adjusted for sex and age)

In order to assess the association between serum vitamin E levels and MS diagnosis, the serum vitamin E levels of patients were compared with those of 94 healthy subjects (n = 49 females) with a mean age of 42.10 ± 14.90 years. The MS patients had significantly lower serum vitamin E levels (MS patients = 24.20 ± 7.45 µmol/L; Control Group = 28.80 ± 9.48 µmol/L, p-value < 0.001). This observation was further confirmed by logistic regression analyses. In the unadjusted model (Model 1) the results showed that serum vitamin E levels were significantly associated with MS diagnosis (OR = 0.93, 95% CI = 0.91–0.97, p-value < 0.001). This model had an AUC of 0.652, indicating a modest ability to discriminate between MS cases and controls. In the adjusted model (Model 2), the association remained almost unchanged (OR = 0.94, 95% CI = 0.92–0.98, p-value < 0.001) with an AUC of 0.679 (Table 2, Fig. 2). Both models remain statistically significant after applying Bonferroni correction (p < 0.0125).

**Table 2:** Logistic regression results for serum vitamin E levels and MS diagnosis

	OR	95% CI	p-value	AUC
Model 1	0.93	0.91-0.97	< 0.001***	0.653
Model 2	0.94	0.92-0.98	< 0.001***	0.678

*OR* Odds Ratio, *CI* 95% Confidence Interval, *AUC* Area Under the Curve. *Model 1* (unadjusted), *Model 2* (Model 1 + age and sex). *** p-value < 0.001

### Cognitive Function

Linear regression analyses indicated a significant association between MMSE scores and serum vitamin E levels in MS patients (Model 1) These associations remained significant after adjusting for potential confounders (Model 2-Model 4) and indicated that higher serum vitamin E levels were associated with better cognitive functioning in MS patients (Table 3). Associations remained statistically significant after Bonferroni correction (p < 0.0125) for Models 2–4, but not for Model 1 (p = 0.013).

**Table 3:** Multiple regression analysis between MMSE scores and serum Vitamin E levels

	β	SE	95% CI	t-Statistic	p-value
Model 1	0.056	0.023	0.011–0.100	2.510	0.013*
Model 2	0.065	0.022	0.025–0.110	2.883	0.005**
Model 3	0.061	0.022	0.016–0.105	2.729	0.007**
Model 4	0.065	0.022	0.020–0.110	2.892	0.004**

β: regression coefficient, SE: Standard Error, CI (95%): 95% Confidence Interval. *Model 1*: Serum Vitamin E levels; *Model 2*: Model 1 + age, sex and education; *Model 3*: Model 2 + disease duration, MS subtype, relapse in the last year, use of disease-modifying drugs; *Model 4*: Model 3 + smoking status, alcohol intake, BMI, hypertension, hyperthyroidism. * p-value < 0.05; **p-value < 0.01

**Fig. 3:**
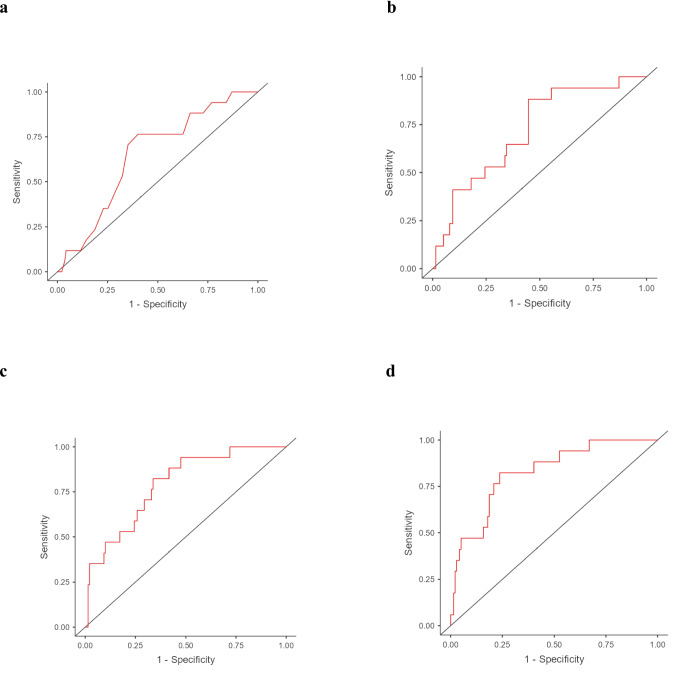
ROC curves for the four logistic regression models assessing the association between serum vitamin E levels and MMSE, with a clinical cut-off of ≤ 24. Panel a shows *Model 1* (unadjusted), Panel b shows *Model 2* (adjusted for age, sex and education), Panel c shows *Model 3* (adjusted for MS-related factors), and Panel d shows *Model 4* (adjusted for factors that could influence serum vitamin E levels)

In particular, a unit increase in Vitamin E was associated to an increase of around 0.065 points in MMSE score considering constant the remaining set of covariates. These results were also confirmed by categorizing patients based on a MMSE cut-off of ≤ 24 (Table S1). The serum vitamin E levels in patients with MMSE ≤ 24 were 21.3 ± 5.58 µmol/L, while those with MMSE > 24 had serum vitamin E levels of 24.6 ± 7.59 µmol/L (p-value = 0.047), indicating a significant difference between the two groups. For *Model 1* (unadjusted), a ten-unit increase in vitamin E was associated with a significant reduction in the risk of cognitive impairment (OR = 0.92, 95% CI = 0.85–1.00, p-value = 0.049), with an AUC of 0.651. In the adjusted models the risk of cognitive impairment remained almost unchanged with a corresponding AUC ranging from 0.720 (*Model 2*) to 0.826 (*Model 4*) (Table S1, Fig. 3).

### Disability

Significant associations were found between EDSS scores and serum vitamin E levels in MS patients. Specifically, linear regression analyses indicated a significant and negative relationship between EDSS scores and serum vitamin E levels in MS patients (Model 1). These associations remained significant after adjusting for potential confounders (Model 2-Model 4) and indicated that higher serum vitamin E levels were associated with a lower degree of disability in MS patients (Table 4). Associations remained statistically significant after Bonferroni correction (p < 0.0125) for Models 2–4, but not for Model 1 (p = 0.028).

**Table 4:** Multiple regression analysis between EDSS scores and serum Vitamin E levels

	β	SE	95% CI	t-Statistic	p-value	B
Model 1	−0.044	0.019	−0.083—−0.004	−2.220	0.028*	−0.169
Model 2	−0.065	0.019	−0.103—−0.027	−3.380	< 0.001***	−0.248
Model 3	−0.049	0.015	−0.081—−0.010	−3.175	0.002**	−0.190
Model 4	−0.051	0.015	−0.082—−0.019	−3.220	0.002**	−0.194

β: regression coefficient, SE: standard error, CI (95%): 95% confidence interval, B: standardized regression coefficient. *Model 1*: Serum Vitamin E levels; *Model 2*: Model 1 + age and sex; *Model 3*: Model 2 + disease duration, MS subtype, relapse in the last year, use of disease-modifying drugs; *Model 4*: Model 3 + smoking status, alcohol intake, BMI, hypertension, hyperthyroidism. * p-value < 0.05; ** p-value < 0.01; *** p-value < 0.001

**Fig. 4:**
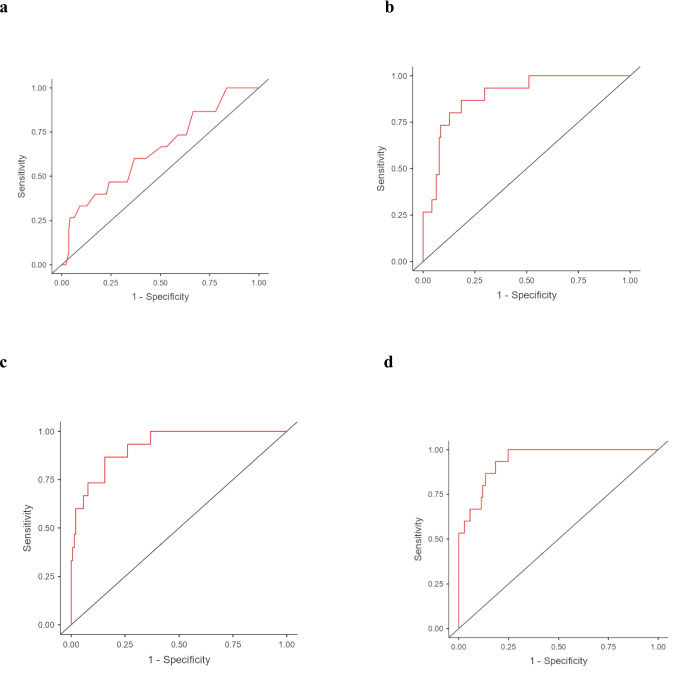
ROC curves for the four logistic regression models assessing the association between serum vitamin E levels and EDSS, with a clinical cut-off of ≥ 6. Panel a shows *Model 1* (unadjusted), Panel b shows *Model 2* (adjusted for age and sex), Panel c shows *Model 3* (adjusted for MS-related factors), and Panel d shows Model 4 (adjusted for factors that could influence serum vitamin E levels)

These results were also confirmed by categorizing patients based on a EDSS clinical cut-off of ≥ 6 (Table S1). In fact, the mean serum vitamin E levels in patients with EDSS < 6 were 24.7 ± 7.45 µmol/L, while those with EDSS ≥ 6 had serum vitamin E levels of 20.3 ± 6.38 µmol/L (p-value = 0.017), showing a significant difference between the two groups. Logistic regression analysis showed that a ten-unit increase in vitamin E was associated with a significant reduction in the risk of higher disability (OR = 0.42, 95% CI = 0.18–0.96, p-value = 0.040, Model 1), with an AUC of 0.651. In the adjusted models the risk of disability remained almost unchanged with a corresponding AUC ranging from 0.893 (*Model 2*) to 0.941 (*Model 4*) (Table S1, Fig. 4).

### Depressive symptoms

Regarding the HDRS, no significant associations were found with serum vitamin E levels across any of the four tested models **(**Table 5**)**. Similar results were obtained using logistic regressions with a cut-off value of ≥ 8 (Table S1).

**Table 5:** Multiple regression analysis between Hamilton Depression Rating Scale and serum Vitamin E levels

	β	SE	95% CI	t-Statistic	p-value	B
Model 1	0.072	0.047	−0.021 – 0.167	1.520	0.131	0.128
Model 2	0.059	0.047	−0.034 – 0.154	1.252	0.213	0.106
Model 3	0.057	0.048	−0.038- 0.154	1.184	0.238	0.102
Model 4	0.053	0.049	−0.043–0.151	1.096	0.275	0.095

β: regression coefficient, SE: Standard Error, CI (95%): 95% Confidence Interval, B: standardized regression coefficient. *Model 1*: Serum Vitamin E levels; *Model 2*: Model 1 + age and sex; *Model 3*: Model 2 + disease duration, MS subtype, relapse in the last year, use of disease-modifying drugs; *Model 4*: Model 3 + smoking status, alcohol intake, BMI, hypertension, hyperthyroidism.

## Discussion

The aim of this study was to explore the associations between serum levels of vitamin E and cognitive function, disability, depressive symptoms, and the MS diagnosis. Our findings showed that MS patients had significantly lower serum vitamin E levels compared to healthy controls, with lower serum vitamin E levels being associated with MS diagnosis. In MS patients, higher serum vitamin E levels were associated with better cognitive function, as measured by MMSE, while lower serum vitamin E levels were associated with higher disability, as indicated by the EDSS score. However, no significant relationships were found between serum vitamin E levels and depressive symptoms, as indicated by HDRS score.

### MS Diagnosis and Serum Vitamin E Levels

Our findings indicate that MS patients had significantly lower serum vitamin E levels compared to healthy controls. This observation is consistent with previous studies, including those by Besler et al. [23] and Jiménez-Jiménez et al. [24], which also reported reduced vitamin E levels in individuals with MS compared to healthy controls [23, 24]. This reinforces the idea that oxidative stress plays a key role in MS pathophysiology as vitamin E is an important antioxidant that protects cells from oxidative damage [21, 22]. We also found that lower serum vitamin E levels were associated with MS diagnosis, and this association remained significant after Bonferroni correction. Lower serum vitamin E levels in MS patients may reflect increased oxidative stress and lipid peroxidation associated with neuroinflammation and demyelination processes characteristic of the disease [21, 22]. While serum vitamin E levels showed modest discriminative ability between MS patients and controls (AUC = 0.678), vitamin E assessment could be integrated into a broader panel of oxidative stress biomarkers to better characterize disease-related metabolic alterations in MS.

### Cognitive Function and Serum Vitamin E Levels

Our study demonstrated a significant positive relationship between serum vitamin E levels and cognitive function in MS patients. Patients with higher serum vitamin E levels had better cognitive performance, as evidenced by higher MMSE score. This finding aligns with previous studies suggesting that vitamin E, an antioxidant, may protect against oxidative stress, a key factor in the pathophysiology of MS and cognitive decline [21, 22]. Oxidative stress has been implicated in the neuronal damage seen in MS, and antioxidants like vitamin E may help mitigate this damage, potentially leading to improved cognitive outcomes. Several studies have highlighted the neuroprotective effects of vitamin E in various neurological disorders and the efficacy of vitamin E supplementation in improving cognitive function [23, 39]. Importantly, the relationship between serum vitamin E levels and MMSE scores remained statistically significant after Bonferroni correction across adjusted models (Models 2–4, all p < 0.0125), even after controlling for demographic, MS-related, and metabolic confounders, demonstrating the robustness of the findings. Furthermore, using a clinical cut-off of MMSE ≤ 24, serum vitamin E levels showed good discriminative ability for identifying cognitive impairment with an AUC of 0.826 in the fully adjusted model, indicating strong potential as a biomarker of cognitive status in MS. These results suggest that vitamin E may play a meaningful role in the context of chronic inflammation and oxidative stress that characterize MS and contribute to cognitive decline.

### Disability and Serum Vitamin E Levels

Our study found a significant negative association between serum vitamin E levels and EDSS scores. Specifically, lower serum vitamin E levels were associated with higher levels of disability. Vitamin E's antioxidant properties may reduce the inflammatory processes that contribute to disease progression and disability in MS. Oxidative damage to the myelin sheaths and neuronal cells in MS could be counteracted by antioxidants like vitamin E, leading to a potential reduction in disability over time. Moreover, studies have shown that higher levels of other antioxidants, such as vitamin D, are linked to lower disability in MS patients [40]. Importantly, the relationship between serum vitamin E levels and EDSS scores remained statistically significant after Bonferroni correction across adjusted models (Models 2–4, all p < 0.0125), even after controlling for demographic, MS-related, and metabolic confounders. Furthermore, using a clinical cut-off of EDSS ≥ 6, serum vitamin E levels showed excellent discriminative ability for identifying patients with higher disability, with an AUC of 0.941 in the fully adjusted model, indicating strong potential as a biomarker of disease severity in MS.

### Depressive symptoms and Serum Vitamin E Levels

Interestingly, no significant associations were found between serum vitamin E levels and depressive symptoms in this study. This lack of association contrasts with some previous studies suggesting a potential link between oxidative stress, antioxidant levels, and mood disorders, including depression, in MS patients [41]. However, the absence of a significant relationship between vitamin E and depressive symptoms in our study may be due to several factors, including the complexity of depression in MS, which may not solely be influenced by oxidative stress. Depression in MS is likely multifactorial, with contributions from biological, psychological, and social factors [9].

## Clinical Implications

The present findings suggest that vitamin E levels may have relevant clinical implications in the management of MS. Higher serum vitamin E levels were associated with better cognitive performance and lower disability, indicating that vitamin E status could represent a potentially useful biological marker to support clinical decision-making and patient monitoring. From a clinical perspective, assessing serum vitamin E levels may help identify patients at greater risk of cognitive impairment and higher disability, who might benefit from closer monitoring and more intensive rehabilitation and cognitive interventions. In this context, vitamin E could be considered as part of a broader panel of biomarkers contributing to a more individualized management of MS. Regarding potential supplementation strategies, previous studies have investigated doses typically ranging from 400 to 2000 IU/day. Guan et al. [42] examined the effects of 400 mg/day of alpha-tocopherol in MS patients over 3 months, reporting significant reductions in serum lipid peroxidation levels and maintenance of telomere length. However, recent systematic reviews indicate that no clinical trials have directly demonstrated that vitamin E supplementation improves cognitive outcomes or reduces disability progression in MS patients [43]. Preclinical studies in experimental autoimmune encephalomyelitis animal models suggest potential benefits of vitamin E supplementation on motor dysfunction and demyelination [44], but human validation is lacking. Given the current lack of clinical trial evidence, vitamin E supplementation cannot be systematically recommended for cognitive or disability outcomes in MS patients at this time. The associations observed in this cross-sectional study require confirmation through prospective longitudinal studies and randomized controlled trials before translating into clinical practice guidelines.

### Limitations and Future Directions

There are several limitations to this study that should be considered. First, the cross-sectional nature of the study limits our ability to infer causal relationships between serum vitamin E levels, cognitive function and disability. Therefore, the relationship observed between serum vitamin E levels and MS diagnosis should be interpreted as associational rather than indicative of causal or prospective risk. Longitudinal studies are needed to determine whether changes in serum vitamin E levels over time are associated with changes in cognitive performance, disability progression, or the onset of MS. Second, the cognitive assessment tool used in this study (MMSE) has recognized limitations in the context of MS. Although MMSE is widely used in clinical practice, it has lower sensitivity compared to MS-specific cognitive batteries such as the Symbol Digit Modalities Test (SDMT) [45] or the Brief International Cognitive Assessment for MS (BICAMS) [46]. Future studies should employ more sensitive and MS-specific cognitive assessment tools to better capture the cognitive deficits characteristic of MS. Third, the modest effect sizes observed (β = 0.065 for cognition, β = −0.051 for disability) suggest that vitamin E represents one component of a complex multifactorial system. Cognitive function and disability in MS are influenced by multiple factors including inflammation, neurodegeneration, genetic factors, disease-modifying therapies, and lifestyle variables. Future research should investigate: (1) whether lower serum vitamin E levels in healthy populations are prospectively associated with increased risk of developing MS through longitudinal studies; (2) whether vitamin E supplementation can provide clinical benefits for cognitive function and disability in MS through randomized controlled trials, including determination of optimal dosing regimens, treatment duration, timing of supplementation initiation (at disease onset, during relapsing–remitting phases, or in progressive MS), and patient selection criteria based on baseline serum vitamin E levels; (3) the long-term safety of vitamin E supplementation, particularly regarding potential interactions with disease-modifying therapies and bleeding risk in patients receiving anticoagulant treatments; (4) the mechanisms underlying the associations between vitamin E and clinical outcomes in MS, including the role of specific oxidative stress pathways and immune modulation. It would also be important to explore the role of vitamin E in combination with other antioxidants or disease-modifying treatments to determine its potential therapeutic benefits.

## Conclusions

In conclusion, our study found that MS patients had significantly lower serum vitamin E levels compared to healthy controls, with lower serum vitamin E levels being associated with MS diagnosis. In MS patients, significant associations were observed between serum vitamin E levels and both cognitive function and disability. Higher serum vitamin E levels were associated with better cognitive performance and lower disability levels. However, no significant associations were found between serum vitamin E levels and depressive symptoms, suggesting that other factors may be more strongly related to mood disorders in MS. These findings indicate that serum vitamin E levels are associated with cognitive performance and disability in MS patients. However, given the cross-sectional design of the study, these results should be interpreted as associative rather than causal. Further research, including prospective longitudinal studies and randomized controlled trials, is needed to better understand the role of vitamin E in MS pathophysiology and to determine whether vitamin E supplementation could provide clinical benefits in this population.

## Supplementary Information

Below is the link to the electronic supplementary material.

Supplementary file1 (DOCX 15.6 KB)

## Data Availability

The datasets generated during and/or analysed during the current study are not publicly available due to ethical restrictions related to patient privacy and the use of retrospective medical records but are available from the corresponding author on reasonable request **.**
