# Supplementary material for: Serum Vitamin E Levels in Multiple Sclerosis: Association with Diagnosis, Cognitive Function, Disability, and Depressive Symptoms
Source: Mol Neurobiol. 2026 Apr 30;63(1):597. doi: 10.1007/s12035-026-05878-w (PMC13132958; doi:10.1007/s12035-026-05878-w)
Supplement: Supplementary file 1 — Supplementary file1 (DOCX 15.6 KB) [file 12035_2026_5878_MOESM1_ESM.docx]

**Serum Vitamin E Levels in Multiple Sclerosis: Impact on Disease Risk, Cognitive Function, Disability, and Depressive Symptoms**

**Molecular Neurobiology**

Francesco **Bruno^1^,** Patrizia **Spadafora^2*^,** Mario Luca **Cuconati^3^,** Antonio **Qualtieri^2^,** Ida **Veltri^4^,** Selene **De Benedittis^2^,** Beatrice Maria **Greco^2^,** Annamaria **Cerantonio^2^,** Luigi **Citrigno^2^,** Gemma **Di Palma^2^,** Olivier **Gallo^2^,** Alberto **Montesanto^5^,** Francesca **Cavalcanti^2^**

**^1^** Department of Human and Social Sciences, Faculty of Social and Communication Sciences, Universitas Mercatorum, Piazza Mattei 10, 00186, Rome, Italy

^2^ Institute for Biomedical Research and Innovation (IRIB), Italian National Research Council (CNR), Loc. Burga 44, 87050 Mangone (CS), Italy

^3^ Student at the Department of Medical and Surgical Sciences, Science and Techniques of Cognitive Psychology Degree Course, Magna Graecia University of Catanzaro, Viale Europa Snc, 88100 Catanzaro, Italy

^4^ Territorial Social-Health Company of Lodi, Piazza Ospitale 10, 26900 Lodi, Italy

^5^ Department of Biology, Ecology and Earth Sciences, University of Calabria, Via Pietro Bucci Snc, 87036 Rende (CS), Italy

***Correspondence:**

Patrizia Spadafora ([patrizia.spadafora@cnr.it](mailto:patrizia.spadafora@cnr.it))

ORCID 0000 0001 5890 4561

**Table S1** Logistic regression results for serum vitamin E levels and MMSE, EDSS and HDRS scores

| **MMSE score** | | | | |
| --- | --- | --- | --- | --- |
| Model 1 | 0.92 | 0.85-1.00 | 0.049* | 0.651 |
| Model 2 | 0.91 | 0.84-0.99 | 0.034* | 0.720 |
| Model 3 | 0.91 | 0.84-0.99 | 0.047* | 0.791 |
| Model 4 | 0.90 | 0.82-0.99 | 0.030* | 0.826 |
|  | | | | |
| **EDSS score** | | | | |
| Model 1 | 1.09 | 1.00-1.19 | 0.038* | 0.652 |
| Model 2 | 1.19 | 1.06-1.33 | 0.002** | 0.875 |
| Model 3 | 1.19 | 1.05-1.34 | 0.006** | 0.904 |
| Model 4 | 1.23 | 1.06-1.42 | 0.005** | 0.924 |
|  | | | | |
| **HDRS score** | | | | |
| Model 1 | 1.02 | 0.11-1.03 | 0.301 | 0.557 |
| Model 2 | 1.04 | 0.97-1.07 | 0.287 | 0.624 |
| Model 3 | 1.02 | 0.97-1.07 | 0.307 | 0.665 |
| Model 4 | 1.02 | 0.96-1.07 | 0.444 | 0.719 |

OR: Odds Ratio, CI = 95% Confidence Interval, AUC: Area Under the Curve. **MMSE score**: Model 1 (unadjusted), Model 2 (Model 1 + sex, age, education), Model 3: Model 2 + disease duration, MS subtype, last year relapse, use of disease modifying drugs, Model 4: Model 3 + smoking status, alcohol intake, BMI, hypertension, hyperthyroidism. **EDSS and HDRS score**: Model 1 (unadjusted), Model 2 (Model 1 + sex, age), Model 3: Model 2 + disease duration, MS subtype, last year relapse, use of disease modifying drugs, Model 4: Model 3 + smoking status, alcohol intake, BMI, hypertension, hyperthyroidism. * p-value < 0.05; ** p-value < 0.01; *** p-value < 0.001.
